# Stiffness and Density Relationships in Additively Manufactured Structures: A Virial Theorem-Based Approach

**DOI:** 10.3390/ma18153432

**Published:** 2025-07-22

**Authors:** Tomáš Stejskal, Silvia Maláková, Marcela Lascsáková, Peter Frankovský

**Affiliations:** 1 Department of Engineeringfor Design of Machines and Transport Equipment, Faculty of Mechanical Engineering, Technical University of Kosice, Letna No. 9, 042 00 Kosice, Slovakia; tomas.stejskal@tuke.sk; 2 Department of Applied Mathematics and Informatics, Faculty of Mechanical Engineering, Technical University of Kosice, Letna No. 9, 042 00 Kosice, Slovakia; marcela.lascsakova@tuke.sk; 3 Department of Applied Mechanics and Mechanical Engineering, Faculty of Mechanical Engineering, Technical University of Kosice, Letna No. 9, 042 00 Kosice, Slovakia; peter.frankovsky@tuke.sk

**Keywords:** virial stability, signal energy, materials modeling, structure stiffness, additive production technologies

## Abstract

Topological optimization uses two main optimization conditions aimed at achieving the maximum stiffness at minimum weight of the loaded object, while not exceeding the allowable stress. This process naturally creates complex structures with varying degrees of density. There is a certain regularity between the density of the structure and stiffness, with the optimal density being related to the golden ratio. This study contributes to materials modeling and their characterization by introducing a mathematical theory related to the virial theorem as a predictive framework for understanding stiffness–density relationships in additively manufactured structures. The definition of virial stability and the methodology for deriving this stability from the kinetic and potential components of a random signal are introduced. The proposed virial-based model offers a generalizable tool for materials characterization, applicable not only to topological optimization but also to broader areas of materials science and advanced manufacturing.

## 1. Introduction

Nowadays, additive manufacturing technologies are increasingly being promoted. Three-dimensional printing allows the creation of complex-shaped structures of products that have optimized parameters. The purpose of topological optimization is to create the desired shapes of components while maintaining the highest possible stiffness at the minimum weight. These two optimization criteria have become the basis of simulation methods for the design of modern machine structures. The first optimization condition is an even distribution of stress in the structure. The second condition is a reduction in weight. A characteristic feature of topological optimization is that products resemble various natural patterns in shape. This connection will be justified in the following text.

The density of structures has a significant impact on the stability of the system under extreme loads. Optimization in terms of structural density is often focused on resistance to damage or absorption of impact energy [1,2,3,4]. The issue of the stability of various biomimicry structures has been addressed in works [5,6,7,8]. These works are focused on finding optimal structures, often by empirical methods. It can be assumed that there could be a third optimization condition for the ideal choice of the density of structures, which would also show the highest degree of stability and rigidity. A theoretical optimization condition is sought.

In this paper, an adequate mathematical theory is derived to support the results obtained by simulation. The thermodynamic theory of gases was used to find the necessary connections.

## 2. Materials and Methods

It is known that the total energy of any mechanical system is given by the sum of the potential and kinetic components. In general, the ratio of these components can be arbitrary. However, if we look at a complex system with random processes, the virial theorem applies, and the ratio of total potential energy to total kinetic energy, simultaneously from all components of the system, is 1:2. This result is surprising, because the cause of this phenomenon is not clear at first glance. The virial theorem was derived for the thermodynamic state of a gas, first published by the German researcher Rudolf Clausius. This discovery follows a certain historical sequence, which is linked to the second half of the 19th century.

Clausius’s most famous statement about the second law of thermodynamics was published in German in 1854 [9] and in English in 1856 [10]. However, the concept of entropy was not defined until several years later. In 1865, Clausius defined entropy [11], and in 1870 he stated the virial theorem, which applied to heat [12].

The second law of thermodynamics states that the direction of energy propagation is always given by the direction of equalization of different energy potentials and never the other way around. The second law of thermodynamics can validate that the entropy of isolated systems left to spontaneous evolution cannot decrease, because they always tend towards a state of thermodynamic equilibrium, where the entropy is highest for a given internal energy [13]. The increase in the combined entropy of a system and its surroundings accounts for the irreversibility of natural processes, often referred to by the concept of the arrow of time [14,15].

The second law of thermodynamics corresponds from a mathematical point of view to the central limit theorem. In probability theory, the central limit theorem states that under appropriate conditions, the distribution of a normalized version of the sample mean converges to the standard normal distribution. The oldest version of this theorem is the de Moivre–Laplace theorem, according to which the normal distribution can be used as an approximation to the binomial distribution [16,17].

The binomial distribution is the basis for the principle of energy propagation in a cloud of gas particles. This consideration provides a basis for investigating whether another statistical tool does not inherently describe other thermodynamic quantities without physical connections. Such a phenomenon was found in the form of the virial theorem. This theorem is presented with a physical example; it is also possible to construct its mathematical form. For comparison, we first present the physical model of the virial theorem.

### Physical Model of the Virial Theorem

The definition of the second law of thermodynamics implies that the total energy in a closed system tends to be distributed evenly throughout the entire volume. If this were not the case, then there would naturally have to be different energy potentials, and the entropy would not reach its maximum value. It is a random process in which the overall ratio of average potential and average kinetic energy stabilizes. Based on this, the virial theorem is a direct consequence of the second law of thermodynamics.

A set of particles of different masses is determined in a potential field by position vectors $r_{i}$ on which forces caused by potential energy and by kinetic energy simultaneously act. Thus, in general, a system of particles and applied forces corresponds to the dot product of momentum and position. Then, the potential quantity G, which generally includes all types of forces, can be defined as

(1) $$G=\sum_{i}p_{i}r_{i}\;,$$

where $p_{i}=m_{i}v_{i}$ is the momentum of the i-th component of the system, and $r_{i}$ is the position vector of the i-th component measured from the origin. The law of conservation of momentum in a closed system applies. The time derivate separates the potential $E_{p}$ and the kinetic energy $E_{k}$ of components [18]:

(2) $$\frac{dG}{dt}=\sum_{i}p_{i}\frac{dr_{i}}{dt}+\sum_{i}\frac{dp_{i}}{dt}r_{i}\;,$$

(3) $$\frac{dG}{dt}=\sum_{i}m_{i}{v_{i}}^{2}+\sum_{i}F_{i}r_{i}\;,$$

(4) $$\frac{dG}{dt}=2E_{k}+E_{p}\;.$$

Equation (4) states that for a very large number of components, an average value can be considered, which determines the average value of both kinetic and potential energy over a sufficiently long time interval. The local components of the energy may change, but the total energy from all components does not change with time τ. To distinguish them from the usual energy notation, average energies are indicated by angle brackets. The energy state can be expressed as follows:

(5) $$\frac{1}{\tau }\int_{0}^{\tau }\frac{dG}{dt}\equiv \langle \frac{dG}{dt}\rangle =2\langle \frac{1}{2}\sum_{i}m_{i}{v_{i}}^{2}\rangle +\langle \sum_{i}F_{i}r_{i}\rangle =\frac{1}{\tau }\left[G\left(\tau \right)-G\left(0\right)\right]\;.$$

The function on the right-hand side of the equation approaches zero for a sufficiently large τ, then

(6) $$2\langle E_{k}\rangle +\langle E_{p}\rangle =0\;.$$

The resulting ratio of energy components can be expressed using a virial proportion in the form

(7) $$\frac{\left|\langle E_{p}\rangle \right|}{\left|-\langle E_{k}\rangle \right|}=2\;.$$

The virial theorem allows one to calculate potential energy from knowledge of kinetic energy alone and vice versa.

## 3. Results

The virial theorem can be derived based on mathematical assumptions. This model is suitable for the design of material structures.

### 3.1. Mathematical Model of the Virial Theorem

**Figure 1:**
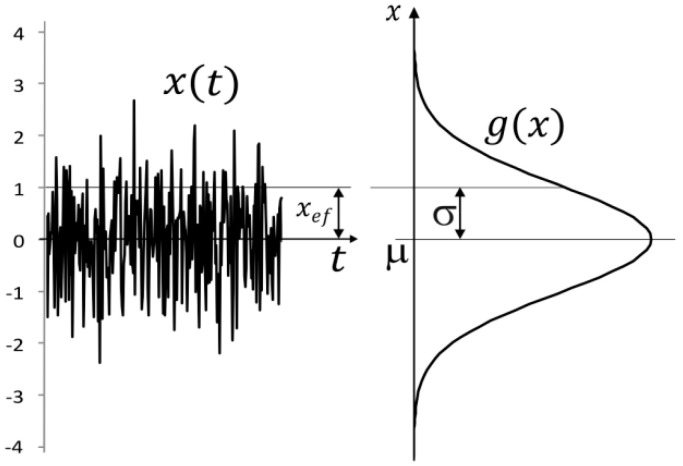
Normal distribution of a white noise signal.

The mathematical model is based only on mathematical assumptions. The starting point is mathematical statistics represented by the binomial distribution of random events. The binomial distribution can describe random physical phenomena, such as the random motion of gas molecules, but it is inherently a concept, independent of the physical world. The mathematical approximation of the binomial distribution to a continuous function is the well-known normal probability distribution. The normal probability distribution for the general parameters of standard deviation  σ and mean μ is defined by a probability density of the form

(8) $$g\left(x\right)=\frac{1}{\sigma \sqrt{2\pi }}e^{-\frac{{\left(x-\mu \right)}^{2}}{2\sigma^{2}}},\;\;\;\;\;\;\;\;\;g\left(x\right)\in \left(0,1\right)\;,$$

where x is the parameter of the random event. Random phenomena can be of a different spatial nature. The simplest manifestation of randomness is a sequence of independent values describing some phenomenon. It could also be a random signal. In a random signal, time is not understood as a continuous physical quantity but as a sequence of normally distributed random events that change the value of the signal. In this way, physical time can be discretized and perceived as a mathematical object without physical meaning. This means that a time unit, for example a second, is given by the order of values. The randomness principle causes the probability distribution of values around the mean value of the signal to be naturally normal (Figure 1).

A random signal has certain specific properties that partly express the relationship to physical models. It is about the power and energy signal.

The power signal is officially defined in the form

(9) $$P_{s}=\underset{T\to \infty }{\lim}\frac{1}{T}\int_{-T/2}^{T/2}x^{2}\left(t\right)dt=x_{ef}^{2}\;,$$

where $x\left(t\right)$ is an arbitrary signal without specifying a physical dimension, $x_{ef}$ is the rms value of the signal, and T is the period of the signal. The rms value of the signal has a normal distribution for white noise, and it is equal to the standard deviation of the distribution.

The energy signal is defined as follows:

(10) $$E_{s}=\int_{-\infty }^{\infty }x^{2}\left(t\right)dt\;.$$

In a steady, stationary signal, the average signal power does not change over a sufficiently long period of time. The above results are presented in signal processing theory [19,20].

In general, signal energy increases continuously over time. Therefore, to characterize the process, the probability of the signal $g\left(x\right)$ occurring depending on the deviation x is used. This probability is proportional to the average power of the signal.

Both the power and signal energy parameters depend on the square of the deviation. Analogously, it can be considered in relation to the normal distribution that there is a power or energy distribution of a random phenomenon in the form of a quadratic function.

The exponentiation of the normal distribution slims down the resulting distribution by a constant ratio $1/\sqrt{2}$.

(11) $$g^{2}\left(x\right)=\frac{1}{\sigma^{2}2\pi }e^{-\frac{{\left(x-\mu \right)}^{2}}{\sigma^{2}}}=\frac{1}{\sigma 2\sqrt{\pi }}\frac{1}{\left(\frac{\sigma }{\sqrt{2}}\right)\sqrt{2\pi }}\;e^{-\frac{{\left(x-\mu \right)}^{2}}{2{\left(\frac{\sigma }{\sqrt{2}}\right)}^{2}}}.$$

We get a new normal distribution with standard deviation $\sigma /\sqrt{2}$. The expression before the exponent only changes the height of the division but does not change the shape.

**Figure 2:**
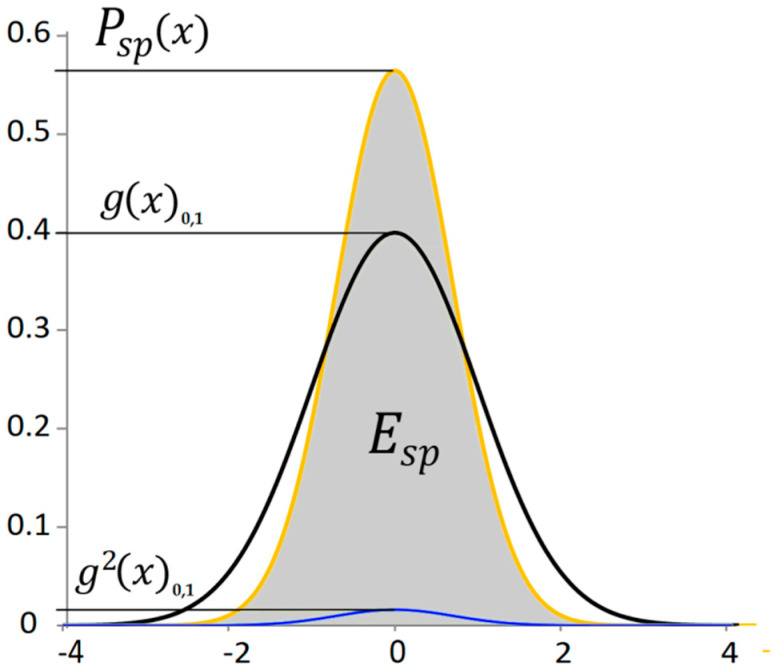
The standard normal distribution of a random signal and the distribution of the potential energy signal.

Let the distance of a point from the mean value of a random signal express the potential energy of the signal. Then the shape of the probability distribution of the potential energy as a function of the deflection x can be obtained by exponentiating the normal distribution. This exponentiating is similar in nature to determining the efficiency of a mechanical system. The total efficiency is given by the product of the efficiencies of the individual components. In this case, it is the probability of reaching a given deviation and the probability of the energy value at a given deviation. Both probabilities have the same distribution. To highlight the probabilistic property of the normal distribution, in the following text we will work with the standard normal distribution ${g\left(x\right)}_{0,1}$. With the standard normal distribution, the displayed curve clearly expresses the probability of the phenomenon. Two identical standard normal distributions are multiplied together. The probability distribution of potential energy is then of the form

(12) $$E_{sp}=k\int_{-\infty }^{\infty }{g^{2}\left(x\right)}_{0,1}dx=\int_{-\infty }^{\infty }P_{sp}\left(x\right)dx=1\;,$$

where $E_{sp}$ is the area proportional to the total potential energy of the signal or the average power from the potential energy, $P_{sp}\left(x\right)$ is the probability of the power of the potential energy of the signal depending on the deviation, ${g\left(x\right)}_{0,1}$ is the standard normal distribution of the signal deviation, and k≈35.4461 is the conversion coefficient that ensures a unit area under the curve $P_{sp}\left(x\right)$ (Figure 2). This probability corresponds in the time domain to the power of the potential energy signal.

**Figure 3:**
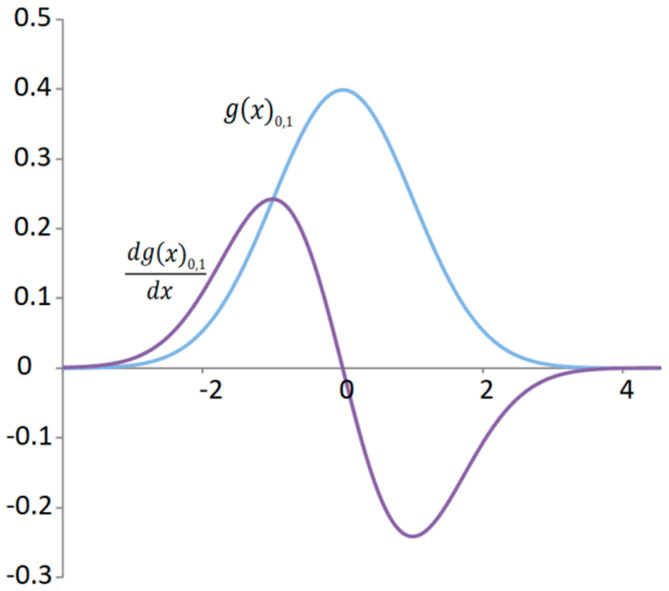
Standard normal distribution of a random deflection signal and distribution of the kinetic energy signal.

In mechanical systems such as a mechanical oscillator, kinetic energy is the derivative of the change in potential energy. In such a simple case, when it comes to the harmonic function of the oscillator’s deflection, the sum of two squared harmonic functions gives a constant. This result of the sum of the potential and kinetic energy corresponds to the total energy, which does not change based on the conservation law of energy. Analogously, it can be established in relation to the normal distribution that the kinetic energy of the signal will have a random distribution obtained by the derivative of the normal distribution (Figure 3), which we will subsequently square.

The procedure for deriving the kinetic energy of a signal is designed based on the analogy with a mechanical oscillator.

**Figure 4:**
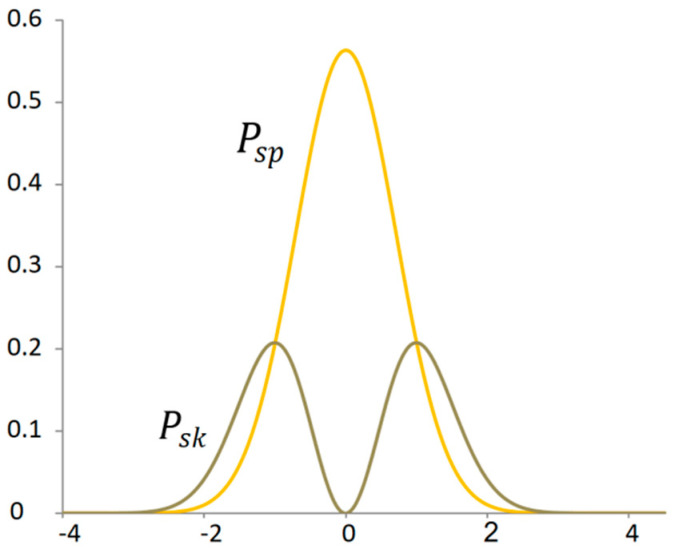
Power distribution of potential and kinetic energy signals.

By exponentiating the derivative of the normal distribution, the distribution of the average kinetic energy output is obtained (Figure 4). Using Equation (12), the area corresponding to the kinetic energy can be determined:

(13) $$E_{sk}=k\int_{-\infty }^{\infty }{\left(\frac{dg{\left(x\right)}_{0,1}}{dx}\right)}^{2}dx=\int_{-\infty }^{\infty }P_{sk}\left(x\right)dx=0.5\;,$$

where $E_{sk}$ is the area proportional to the total kinetic energy of the signal or the average power from the kinetic energy, k≈3.54461 is the conversion coefficient, and $P_{sk}\left(x\right)$ is the probability of the power of the kinetic energy of the signal depending on the deflection. That is to say, it is the kinetic power signal. This probability corresponds in the time domain to the power of the kinetic signal energy. In this case, the same conversion coefficient as for potential energy must be used.

**Remark** **1.** *The value of the conversion coefficient* k *does not matter. If any coefficient is used, the ratio of areas under the curves does not change.*

The virial proportion is the ratio of the potential and kinetic energy of the entire random signal equal to two. The virial theorem, from a mathematical point of view, arises by comparing the areas of probability distributions that correspond to the total potential and total kinetic energy of a signal.

(14) $$\frac{E_{sp}}{E_{sk}}=2\;.$$

This result is a specific property of the normal distribution, which is a mathematical concept. The result supports the validity of the shape of kinetic energy but is not direct evidence of it. An area ratio equal to two can theoretically exhibit different curve shapes. On the other hand, the derivative of the normal distribution is the natural shape of the curve. In the following text, it is shown that this shape of the curve is consistent with the Maxwell–Boltzmann distribution of the velocity of molecules of an ideal gas, which supports the correctness of the assumption.

**Remark** **2.** *The area calculation process can be verified numerically. It is therefore appropriate to show numerically that with an increasing accuracy of curve integration, the result of the virial proportion converges to the number two (Table 1).*

### 3.2. Concept of the Virial Theorem for Mechanical Systems with Random Motion

The concept of random molecular motion can also be transferred to compact mechanical systems. Mechanical systems can be understood as various connected oscillators. Mutually independent oscillators that have the same oscillation period creates a random system. The signal will be given by the deviation of the mass point from the equilibrium position. These are mechanical oscillators that are unsteady and have a random distribution of deviations over time. This physical model can be rewritten into a mathematical model by recording each oscillator at a given random position as a sequence of random deflections. The result will be a signal with an arbitrary probability distribution.

For completely independent oscillators, an arbitrary probability distribution can be proposed. In practice, there are only dependent oscillators that together form a complex system. In this case, we are dealing with coupled oscillators, where one oscillator is always the driving oscillator, and the other is the driven oscillator. During the energy transfer, there is a quarter phase shift of the oscillations between the driving and driven oscillators. After all the energy has been relocated from one oscillator to the other, the roles are reversed. In the long term, with a large number of oscillators, the same principle can be considered as in the collisions of gas molecules. Then the energy distribution of the oscillators involved in creating the maximum potential energy will have a normal distribution, and the virial theorem can be applied. The theory of the energy transfer of coupled oscillators is well described in the relevant literature [21,22,23,24].

It is worth noting that simple systems such as an isolated oscillator exhibit a virial ratio of 1:1, because there is no influence of randomness in such a system.

### 3.3. Comparison of the Maxwell–Boltzmann Distribution with the Kinetic Power Signal

A closed system with material objects is subject to gravitational potential energy. The role of potential energy is to hold material objects in a given space. Mass particles must also have kinetic energy. Otherwise, all objects would immediately fall into a common center of gravity, and the motion would cease.

In the case of a gas observed on Earth, only kinetic energy is dominant, which is directly dependent on the square of the molecular velocity. To prevent molecules from escaping, a closed space is necessary. The material walls of an enclosed space represent potential energy. The manifestation of potential energy is then expressed by the gas pressure.

Maxwell was the first to formulate the probability distribution of the velocity of a complex mechanical system [25,26]. The random movement of molecules creates pressure when they collide with a wall. At any moment, some molecules increase the force exerted, while others decrease the force and leave the wall. There are also differences in the speed of impact and the magnitude of the force impulse. We assume that the principle of the central limit theorem applies, and, based on many partial influences, none of which is dominant, a normal distribution of the contribution of individual molecules to the total pressure is realized. Based on this, the potential energy of molecules has a normal distribution.

For the molecules’ speed, two opposing conditions can be defined, determining the final state of the system:

1. The distribution of molecular speeds follow the normal distribution $g\left(x\right)$, where zero speed is the most probable. At zero speed, the potential energy of the molecule is at its maximum. This phenomenon occurs at the moment of collision of two molecules, when maximum pressure is created.
2. The distribution of molecular velocity is subject to a quadratic function $f\left(x\right)=x^{2}$, whose minimum is at zero velocity, which is a characteristic of kinetic energy as a function of velocity’s growth. At the moment of collision of two molecules, the kinetic energy of the molecule under consideration is naturally zero.

The resulting state of the probability distribution of the velocity of molecules in one orientation is given by the product of the above contradictory conditions. The result corresponds to the Maxwell–Boltzmann distribution of molecular velocity.

The parametric expression of the probability density of the Maxwell–Boltzmann distribution is given in the form

(15) $$m\left(x\right)=\frac{1}{a^{3}}\sqrt{\frac{2}{\pi }}x^{2}e^{-\frac{1}{2a^{2}}{\;x}^{2}},\;\;x\in \left(0,\infty \right)\;,$$

where a is the partition parameter depending on the temperature of the ideal gas. After substituting physical units, we have

(16) x=v 

(17) $$a=\sqrt{\frac{k_{B}T}{m}}$$

where v is the velocity of the molecules, $k_{B}$ is the Boltzmann constant, T is the temperature of the gas, and m is the mass of the molecules.

**Figure 5:**
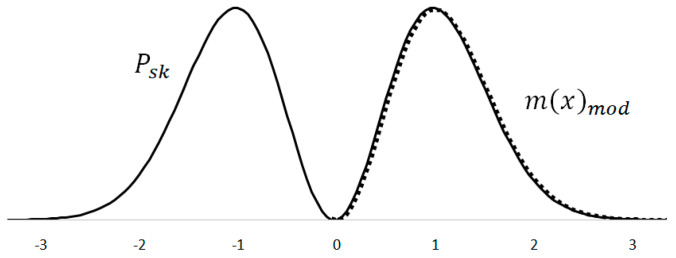
Overlapping of the Maxwell–Boltzmann distribution and the square of the derivative normal distribution.

The kinetic energy performance probability curve $P_{sk}$ is very close in shape to the Maxwell–Boltzmann distribution $m\left(x\right)$ (Figure 5). A numerical comparison can be made by slimming down the Maxwell–Boltzmann distribution ${m\left(x\right)}_{mod}$ using the standard deviation $\sigma =1/\sqrt{2}$. The obtained distribution is adjusted in height by multiplying it with a correction coefficient z=10, thus obtaining a good overlap with the kinetic energy power probability curve. The value of the correction coefficient allows one to achieve the best overlap of the two curves, but at the same time it does not affect the shape of the curves. During the analysis, the emphasis is placed on achieving a good shape matching of the curves.

The resulting correlation of the curve shape is quite high. For 82 numerical values that correspond to the 3 sigma interval, the correlation coefficient is ρ=0.9987. The good shape match supports the theory that the kinetic component of the signal energy can also well describe the probability distribution of the molecular velocity.

### 3.4. Virial Stability of a Random System

Any mechanical system with cyclic or chaotic motion can theoretically have any ratio of kinetic and potential energy. For a random system, the virial theorem was derived, according to which although the components may have different ratios of kinetic and potential energy, the average ratio of all components of the system in the long term will be 2:1. This is a law that speaks about a property of the whole, but it says nothing about the statistical distribution of kinetic energy of this property in space. From the analysis it is clear that in the chaotic motion of gas molecules all states lie between two extreme states. At the zero value of the distribution, there is an extreme probability in which the molecules are not moving and have a maximum potential energy. At the other extreme of the distribution, molecules move at maximum speed, have maximum kinetic energy, and minimum potential energy. Statistically, only a minimal number of molecules are found at both extremes. Most molecules are in a state between these two extremes.

**Definition** **1.** *The virial stability of a random system is the point at which the mode of the kinetic energy distribution of the system has exactly the equality of the probabilities of the potential and kinetic energy outputs.*

Every uncontrolled mechanical system tries to assume the most stable position of its components. The virial theorem expresses this thesis in mathematical language. The most probable state is given by virial stability. The disruption of virial stability can be imagined as the moment when a warm volume of gas mixes with a cold one. Both gas volumes were in virial stability before mixing. A new virial stability occurs only after the temperatures of the mixed gases equalize.

### 3.5. Proportionality of the Virial Theorem

**Figure 6:**
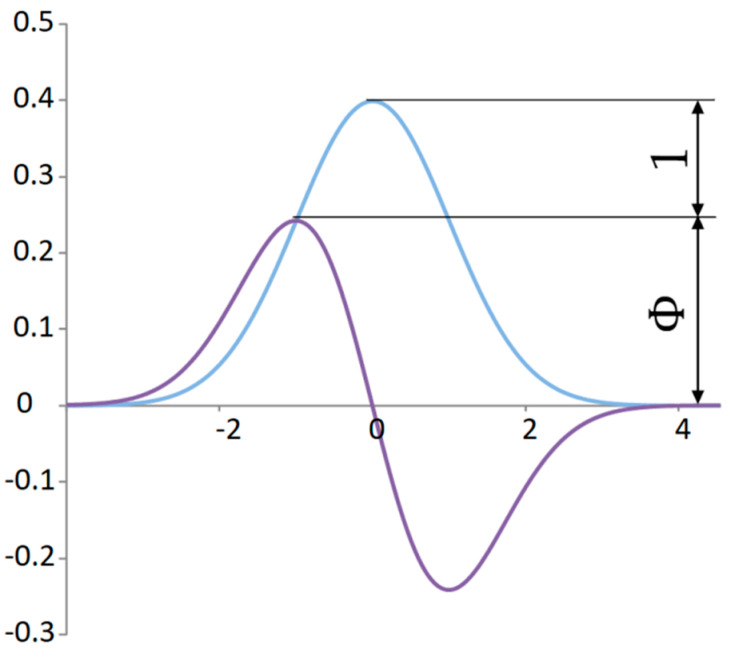
The proportionality between the standard normal distribution and its derivative.

For the proportionality analysis of the normal distribution, it is convenient to compare significant points with the derivative curve in one graph (Figure 6). The ratio of the peaks of the maximum values of random distributions is quite close to the ratio of the golden ratio 1:Φ for Φ≈1.618.

**Figure 7:**
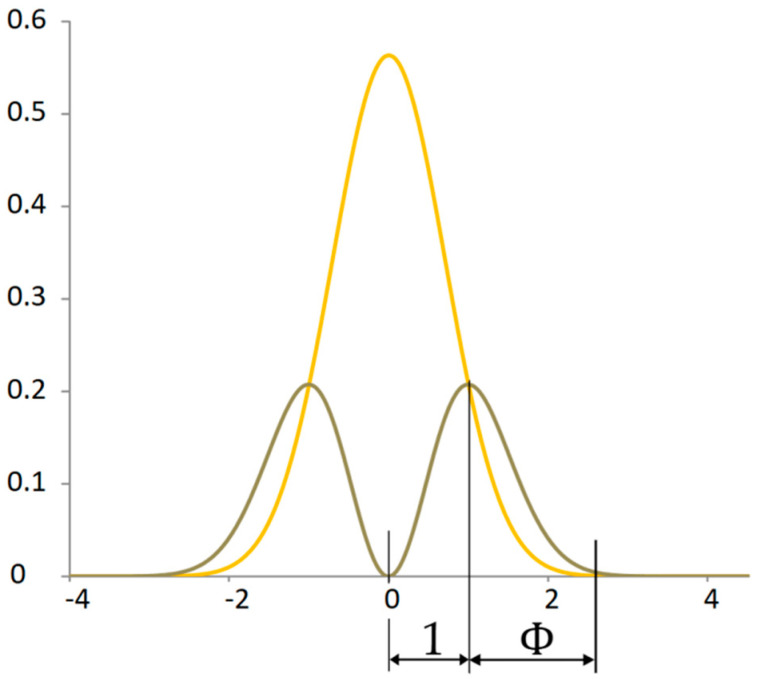
Proportionality between probability distribution of potential and kinetic power signals.

The point that determines the division in the golden ratio is not only the mode of the power distribution of the kinetic energy of the signal but also the intersection of the power distribution with the potential energy of the signal (Figure 7). This dividing point has a specific property. At this point, the increment of the original function is equal to the derivative of the original function. This point expresses virial stability.

It can be stated that at the point of virial stability, the component of the system is equally far from the state of complete immobility as from the state of maximum mobility. If the least probable states of the system represent extreme states, then the most probable state lies in the golden ratio between the two extremes.

### 3.6. Design of Random Material Structures for 3D Printing

Homogeneous structures essentially have two variable parameters.

The first parameter is the arrangement of the components by size. This arrangement corresponds to a geometric series of component sizes. The series can be modified by changing the quotient of the series or by omitting some components.

The second parameter is the rate of components. From an energetic point of view, the most likely relationship is an inverse relationship between the size of a component and its rate in the structure.

Nature always creates the most efficient structures. These structures have the form of a fractal. This is a natural law that is connected to the second law of thermodynamics. The essence is that the distribution of energy to create a structure is influenced by the size of the component and its rate. The analysis shows that the distribution of dynamic elements is ideally in the form of the kinetic power of the signal. This is essentially the derivative of the square of the normal distribution. If the horizontal axis is the size of the components, then the vertical axis will present the ideal rate of elements in the structure. The highest rate is at the point of virial stability.

It can be assumed that deviation from these ideal parameters will create structures with worse properties under load. At a constant volume of mass, which is the structure, this will be reflected in a reduced stiffness compared to a structure with ideal parameters.

**Figure 8:**
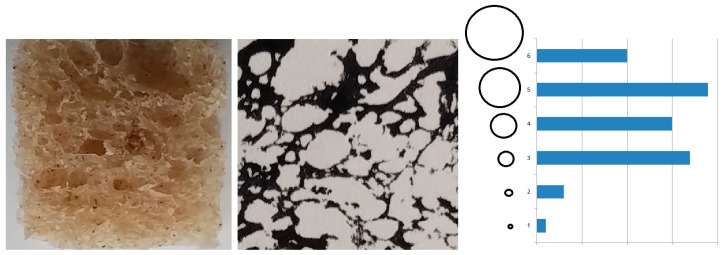
Analysis of the number of holes in a cross-section of bread.

The inspiration for this claim is an analysis of a cross-section of sourdough bread (Figure 8).

Such a random structure can be both analyzed and designed for 3D printing objects.

## 4. Conclusions

The novelty of this paper is its reference to the mathematical description of the virial, which is independent of physics. This suggests that it is a general principle that applies in various fields of technology. The principle generally also applies to the structure of materials with a random distribution of building elements. In the process of manufacturing the structure, dynamic forces act, which are related to kinetic energy. The ideal distribution of the size and frequency of the elements of the structure will be subject to this law. This can be advantageously used in the design of 3D printing as an optimization condition.

## Figures and Tables

**Table 1:** Convergence of the virial proportion, which is dependent on the integration step size.

Step Size	Virial Proportion
0.5	2.0631
0.2	2.0100
0.1	2.0025
0.05	2.0006

## Data Availability

The original contributions presented in this study are included in the article. Further inquiries can be directed to the corresponding author.
